# Thermogalvanic hydrogel-based e-skin for self-powered on-body dual-modal temperature and strain sensing

**DOI:** 10.1038/s41378-024-00693-6

**Published:** 2024-04-28

**Authors:** Zhaosu Wang, Ning Li, Xinru Yang, Zhiyi Zhang, Hulin Zhang, Xiaojing Cui

**Affiliations:** 1 College of Electronic Information and Optical Engineering, Taiyuan University of Technology, Taiyuan, 030024 China; 2 College of Materials Science and Engineering, Taiyuan University of Technology, Taiyuan, 030024 China; 3 School of Physics and Information Engineering, Shanxi Normal University, Taiyuan, 030031 China

**Keywords:** Electrical and electronic engineering, Electronic properties and materials

## Abstract

Sensing of both temperature and strain is crucial for various diagnostic and therapeutic purposes. Here, we present a novel hydrogel-based electronic skin (e-skin) capable of dual-mode sensing of temperature and strain. The thermocouple ion selected for this study is the iodine/triiodide (I^−^/I_3_^−^) redox couple, which is a common component in everyday disinfectants. By leveraging the thermoelectric conversion in conjunction with the inherent piezoresistive effect of a gel electrolyte, self-powered sensing is achieved by utilizing the temperature difference between the human body and the external environment. The composite hydrogels synthesized from polyvinyl alcohol (PVA) monomers using a simple freeze‒thaw method exhibit remarkable flexibility, extensibility, and adaptability to human tissue. The incorporation of zwitterions further augments the resistance of the hydrogel to dehydration and low temperatures, allowing maintenance of more than 90% of its weight after 48 h in the air. Given its robust thermal current response, the hydrogel was encapsulated and then integrated onto various areas of the human body, including the cheeks, fingers, and elbows. Furthermore, the detection of the head-down state and the monitoring of foot movements demonstrate the promising application of the hydrogel in supervising the neck posture of sedentary office workers and the activity status. The successful demonstration of self-powered on-body temperature and strain sensing opens up new possibilities for wearable intelligent electronics and robotics.

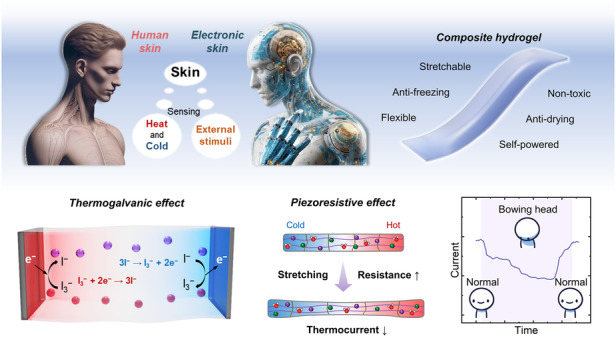

## Introduction

With the ever-evolving landscape of wearable technologies and smart devices, the development of flexible and stretchable electronic skin (e-skin) has emerged as a pioneering field with profound implications for human‒machine interfaces^1,2^, healthcare^3–6^, and robotics^7,8^. E-skin can convert external mechanical or thermal stimuli into electrical signals^9–15^, but the detection of only a single parameter is no longer satisfactory. Instead, the development trend of e-skin is gradually moving toward multimodal detection^16–20^. Meanwhile, the quest for flexible and stretchable electronic skin stems from the realization that traditional rigid electronics are ill suited for applications requiring conformability and adaptability to complex and dynamic surfaces. With its remarkable flexibility and sensitivity, human skin serves as a source of inspiration for the design and development of electronic skin^21^.

Among the various functionalities sought for advanced e-skin, the capability for self-powered sensing stands out as a key research focus^22–24^. The utilization of a temperature difference for self-powering represents a sustainable and efficient approach to enhance the autonomy of electronic skin devices. Li et al. combined the thermoelectric material graphene with Ecoflex to fabricate a one-dimensional self-powered strain sensor^25^. However, its Seebeck coefficient is not high, reaching only a few tens of microvolts per Kelvin. Kwon et al. achieved a certain improvement in the Seebeck coefficient by using a thermoelectric fabric based on bismuth telluride (Bi_2_Te_3_), demonstrating its application as a pressure- and temperature-sensing array system^26^. Nevertheless, the Seebeck coefficients of the mentioned materials are still in the microvolt per Kelvin range. As a class of polymers renowned for their water-absorbing properties and biocompatibility, hydrogels have gained prominence in various biomedical applications. Their ability to retain a high water content, similar to human tissue, renders them an ideal candidate for electronic skin^27–29^. An increasing number of ion-conductive hydrogels have achieved heat-to-electrical energy conversion, depending on the thermodiffusion effect^30,31^, the thermogalvanic effect^32,33^, and synergistic effects^34^. Based on the abovementioned skin-like gels, significant progress has also been made in temperature^35^, pressure^36,37^, and strain sensing^38,39^. Fu et al. developed a tactile sensor with temperature and pressure responsiveness using a thermoelectrochemical hydrogel elastomer, and its equivalent Seebeck coefficient reached millivolts per Kelvin^40^. However, in the application of this elastomer, only a response to pressure from vertical compression (applied from above and below) was demonstrated, without exploring its ability to sense strain in the lateral direction on the human skin surface.

In this study, we engineered a hydrogel-based electronic skin with dual-mode temperature- and strain-sensing capabilities that harvests thermal energy from human body heat. We selected the iodine/triiodide (I^−^/I_3_^−^) redox couple, which is widely employed in disinfectants for various applications, including disinfection of skin, surgical instruments, and medical devices. The composite hydrogel, made from PVA monomers using a simple freeze-thaw method, exhibits flexibility, stretchability, and skin adaptability. The microscale pores within the hydrogel facilitate the transport and reaction of redox couple ions. Betaine was introduced, which enhances the resistance of the hydrogel to dehydration and low temperatures due to the hydrogen bond interactions between zwitterions and water molecules. The resulting thermogalvanic hydrogel exhibits favorable characteristics, including good Seebeck coefficient and conductivity. By combining the thermogalvanic and piezoresistive effects, self-powered temperature and strain sensing are successfully demonstrated through encapsulation and integration of the hydrogels onto human tissue. The self-powered sensing implementation contributes to advancement of multimodal sensing electronic skin in various fields, including healthcare, motion monitoring, virtual reality, and beyond.

## Materials and methods

### Materials

PVA (molecular weight, 120,000 to 140,000), betaine (MW = 117.15, ≥ 98%), I_2_ (MW = 253.81, ≥ 99%), and KI (MW = 166, ≥ 99%) were purchased from MACKLIN Reagent. All materials were used as received without further purification.

### Preparation of thermogalvanic hydrogels

First, different concentrations of I_2_ and KI were added to deionized water and stirred for 1 h at room temperature, and then, a certain amount of betaine was added and stirred until it was completely dissolved to obtain a betaine-I^−^/I_3_^−^ solution. Second, a certain amount of PVA was added to deionized water and stirred at 105 °C for 1 h to obtain a PVA solution. The mass ratio of PVA to total deionized water was fixed at 1:9. Then, the previous solution was poured into the PVA solution. After being stirred at 95 °C for 2 h, the mixed solution was poured into a PTFE mold. Finally, the sample was frozen at −20 °C for 12 h and thawed at 20 °C for gelation.

### Characterization and performance tests

SEM (Hitachi SU8010) was utilized to characterize the morphology of the hydrogels. The tensile and compressive mechanical properties of the hydrogels were measured using a universal mechanical test machine (QT-1196) at room temperature. The tensile test was conducted on a dumbbell-shaped sample at 100 mm·min^−1^. The compressive test was conducted on a cylindrical sample (20 mm in height, 16 mm in diameter) at 5 mm·min^−1^.

### Thermoelectric performance measurement

The output voltage and current were measured by a Keithley 2400 instrument. The temperatures of two Peltier chips were controlled by a direct current source and recorded by thermocouples (NAPUI TR230X). An electrochemical workstation (CHI660e, Shanghai Chenhua Instrument Co. Ltd.) was used to measure the electrochemical impedance spectroscopy (EIS), linear sweep voltammetry (LSV), and cyclic voltammetry (CV) data of the hydrogels. The ionic conductivity was calculated according to σ = d/RA, where d, R, and A represent the thickness, impedance and contact area of the hydrogel, respectively. A flexible electronic tester (FT2000, Shanghai Mifang Electronic Technology Co. Ltd.) was used to quantitatively control the strain of the hydrogel sheets.

## Results and discussion

**Fig. 1:**
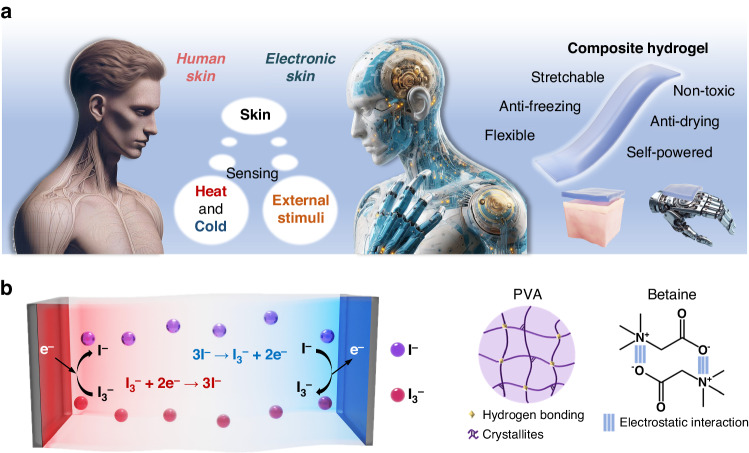
Conceptual schematic diagram of the thermogalvanic hydrogel patch resembling skin for self-powered sensing of temperature and strain. **a** The designed hydrogel functions similar to skin, perceiving temperature and touch. **b** Demonstration of the thermoelectric conversion mechanism and composition of the hydrogels

Figure 1a illustrates the conceptual design of the hydrogel. Both human skin and the electronic skin inherently possess fundamental sensing abilities, such as perceiving external temperature and tactile stimuli. The hydrogel, which combines thermoelectric and piezoresistive effects, can generate electricity by leveraging temperature differences and can detect external tactile stimuli through changes in the electrical resistance induced by deformation. The designed hydrogel offers advantages such as flexibility, stretchability, and resistance to drying, making it suitable for integration onto human skin or machinery for self-powered sensing. The working mechanism of the temperature difference-to-electricity conversion process is shown in Fig. 1b. When a temperature gradient is applied, the temperature-dependent nature of the redox reactions leads to oxidation of the redox couple at the anode and reduction at the cathode. At the anode electrode, due to the low temperature of the oxidation reaction 3I^−^ → I_3_^−^ + 2e^−^, electrons are injected into the cold electrode, increasing its electrochemical potential and causing a decrease in the electrode potential. At the cathode electrode, the favorable thermodynamics for the reduction reaction I_3_^− ^+ 2e^−^ → 3I^−^ at high temperatures attract electrons to the hot electrode, lowering its electrochemical potential and resulting in a higher electrode potential (Fig. S1). Subsequently, the iodine ions generated by the reduction reaction migrate to the low-temperature electrode through convection, diffusion, and migration, while the triiodide ions produced by the oxidation reaction return to the high-temperature electrode, establishing a continuous cyclic reaction. Therefore, a continuous electric current can be generated between the two electrodes under a temperature gradient. For the quasi-solid polymer network, we opted for PVA as the primary monomer and conducted polymerization through a freeze‒thaw method. PVA chains can uniformly crystallize in the hydrogel. Electrostatic interactions exist between the added betaine molecules. In addition, the zwitterions in betaine can form hydrogen bonds with water molecules, thereby enhancing the water retention and anti-freezing properties of the gel. The resulting composite hydrogel features numerous micron-sized pores that facilitate the unrestricted movement of ions (Fig. S2).

**Fig. 2:**
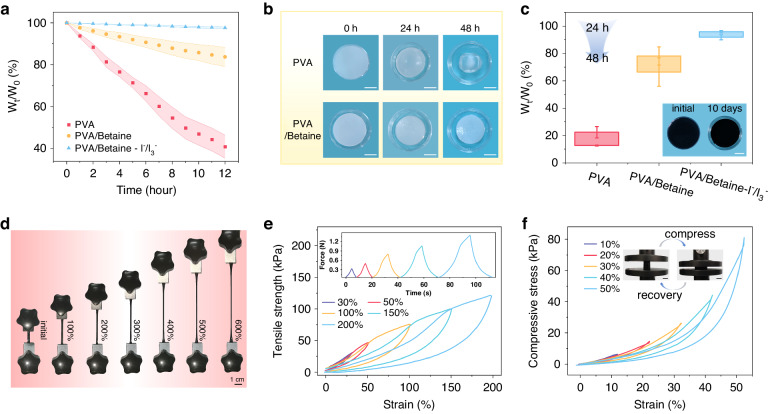
Testing of the anti-dehydration and mechanical properties of the composite gels. **a** Dehydration of three hydrogels over 12 h. **b** Photographs of PVA hydrogels with and without betaine after 24 and 48 h. Scale bar: 1 cm. **c** Weight changes of the three hydrogels between 24 and 48 h in air. Inset: Photograph of the PVA/betaine-I^−^/I_3_^−^ hydrogel after being left at room temperature for ten days. Scale bar: 1 cm. **d** Physical photographs of a PVA/betaine-I^−^/I_3_^−^ hydrogel stretched to 100%, 200%, 300%, 400%, 500%, and 600% of its original length. Scale bar: 1 cm. **e** Loading‒unloading tests for the composite hydrogel with different strains. **f** Compressive stress‒strain curves of the composite gel at 10%, 20%, 30%, 40%, and 50% compressive strains. Inset: Physical photographs of a cylindrical gel during compression testing. Scale bar: 1 cm

We explored the dehydration characteristics of a pure polyvinyl alcohol (PVA) hydrogel and PVA hydrogels with betaine at ambient temperature. Notably, when the PVA-to-betaine mass ratio is 2:5, the composite hydrogel retains more than 80% of its original weight after storage for 12 h (Fig. 2a). Moreover, the presence of the I^−^/I_3_^−^ redox couple increases the water retention capacity of the hydrogel. The incorporation of betaine substantially improves the water retention capability of the PVA hydrogel, which is attributed to the hydrogen bonding between betaine and water. As illustrated in Fig. 2b, after 48 h, the pure PVA hydrogel exhibits significant desiccation and shrinkage, whereas the betaine-supplemented sample experiences only modest shrinkage. The dehydration trends of the three gel samples during the 24–48 h period are depicted in Fig. 2c, revealing that the introduction of betaine enables the gel to retain more than 60% of its original weight after two days. The inset of Fig. 2c demonstrates that the PVA/betaine hydrogel containing I^−^/I_3_^−^ ions maintains a moist gel state even after ten days, with minimal alterations in shape. We conducted mechanical performance tests on the selectively optimized, water-retaining hydrogel. Figure 2d shows the ability of the gel to be consistently stretched to 600% of its original length. The hysteresis loop in Fig. 2e signifies effective energy dissipation within the hydrogel network structure. As the strain intensifies, more of the hydrogel networks are disrupted, facilitating dissipation of the fracture energy. Figure 2f illustrates the compression testing of the hydrogel, with the inset highlighting the good elasticity of the hydrogel when compressed with a mechanical test machine. The tensile cycling test results are shown in Fig. S3. Additionally, Fig. S4 illustrates the process of pressing the hydrogel with a steel ruler, demonstrating the robust recovery of the hydrogel even when subjected to pressure against a sharp edge. Fig. S5 shows photographs of a hydrogel sheet when it is bent, twisted, knotted and stretched, indicating that it has excellent flexibility and stretchability.

**Fig. 3:**
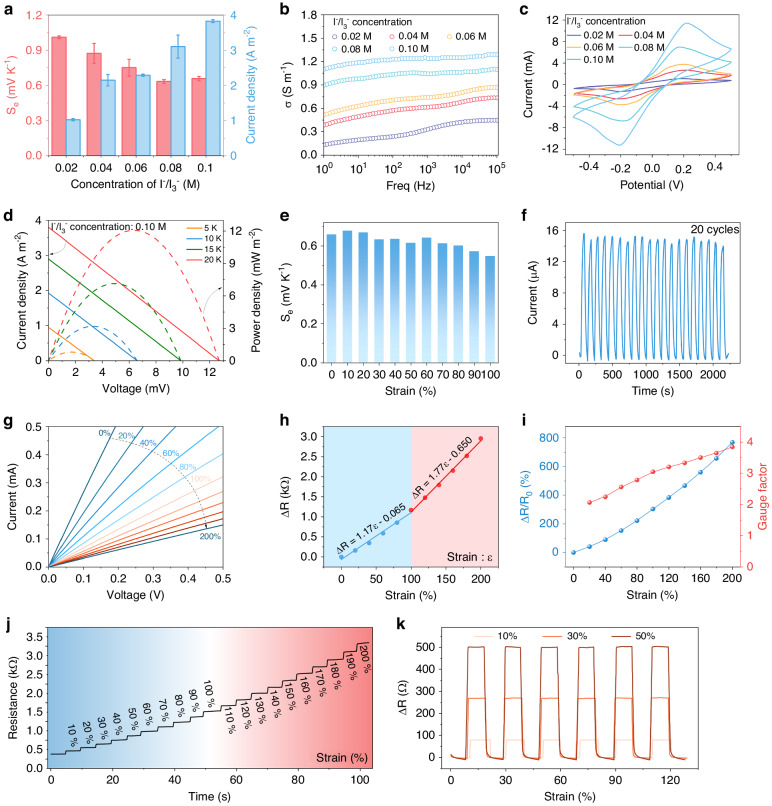
Thermoelectric output performance and resistance change characteristics of the thermogalvanic hydrogels. **a** Thermopower and current density, **b** conductivity, and **c** cyclic voltammograms of PVA/GEL-GL gels with different I^−^/I_3_^−^ concentrations. **d** Power density‒ and current density‒voltage curves for the thermogalvanic hydrogel with a 0.10 M I^−^/I_3_^−^ concentration at different ΔT values. **e** Seebeck coefficients of hydrogels at different tensile strains. **f** Current output of the hydrogel over 20 cycles of an applied temperature difference. **g** Current‒voltage curves of the hydrogel at different stretching strains. **h** Resistance variation of the hydrogel with increasing stretching strain. **i** Relative variation in the resistance and gauge factor with increasing strain from 0% to 200%. **j** Resistance-time relationship at different tensile strains. **k** Stability test for the resistance change

Benefiting from the well-balanced attributes, including anti-drying properties, a thermoelectric output, and mechanical stretchability, the hydrogel comprising PVA and betaine at a mass ratio of 2:5 emerges as the optimal candidate for thermogalvanic conversion. The thermogalvanic gel operates through charge migration between the electrodes of the redox pair, which is driven by entropic changes resulting from temperature differences. This phenomenon manifests as a thermovoltage output at both ends of the electrodes, which is quantified by the Seebeck coefficient (S_e_). Previous studies have consistently highlighted the pivotal role of the redox couple concentration in the energy conversion efficiency. Consequently, our initial investigation aimed to scrutinize the influence of the I^−^/I_3_^−^ concentration on the thermoelectric performance. The output of the thermogalvanic hydrogels was evaluated on a meticulously constructed temperature gradient platform, as shown in Fig. S6. With increasing concentration of redox pair ions, a discernible decrease in the thermovoltage coupled with an increase in the thermocurrent is evident (Figs. 3a, S7). Given our primary emphasis on the current response for subsequent applications, a sample with a redox pair concentration of 0.10 M was deliberately chosen for further experiments to explore self-powered sensing applications. In addition, the voltage output curves under various temperature differences for samples with a redox pair concentration of 0.10 M are shown in Fig. S8. This test method was employed to assess the Seebeck coefficient of samples with different concentrations of redox pairs. The gel conductivity was meticulously assessed using an electrochemical workstation, revealing a direct correlation between the redox ion concentration and gel conductivity (Fig. 3b). This correlation substantiates the earlier finding that the gel manifests its highest current output at the peak I^−^/I_3_^−^ ion concentration. Cyclic voltammograms of the thermogalvanic gels with various I^−^/I_3_^−^ concentrations are presented in Fig. 3c. The symmetry and peak height of the oxidation and reduction waves in the cyclic voltammetry curves offer insights into the reversibility of the redox reaction. Clearly, the observed symmetry in the curves attests to the reversible nature of the redox reaction in the prepared gels. Figure 3d shows the current/power density–voltage curves of the hydrogel with a 0.10 M I^−^/I_3_^−^ concentration recorded under different temperature differences (ΔT) with the cold-end temperature (T_c_) set at 293 K. As ΔT increases from 5 to 20 K, both the open-circuit voltage (V_oc_) and short-circuit current density gradually increase, from 3.40 mV and 0.95 A m^−2^ to 12.60 mV and 3.80 A m^−2^, respectively. Analogous current/power density–voltage curves for other I^−^/I_3_^−^ concentrations can be found in Fig. S9. In light of the thermoelectric foundation integral to the subsequent self-powered strain sensing application utilizing the gel, the thermoelectric output stability under diverse tensile strains was evaluated. Figure 3e illustrates the examination of the thermopower of the gel across different strains. Notably, the Seebeck coefficient exhibits fluctuations commensurate with strain increments, but the overall decay is marginal. Remarkably, even at a strain level of 100%, the Seebeck coefficient retains over 80% of its initial value. This observation underscores the robust thermoelectric response characteristics of the gel under strained conditions. The cycling characteristics of the thermal current were assessed, revealing that the gel has a relatively stable output current over 20 cycles (Fig. 3f). To elucidate the stretching capability of the gel sheet and its strain-sensing ability predicated on resistance variations, the gel was stretched from 0 to 200%. Here, strain is defined as the ratio of the stretched length to the initial length. The corresponding current-voltage (I-V) curve for the stretching strain is depicted in Fig. 3g. With increasing tensile strain, the I-V curve slope decreases, signifying a gradual increase in the resistance. The controlled strain platform employed in the aforementioned experiments is presented in Fig. S10. The corresponding resistance-strain curve exhibits a consistent increase from 0% to 200% strain (Fig. 3h), underscoring the potential of the hydrogel as a strain gauge for sensing applications. Additionally, we calculated the resistance variation [(R − R_0_)/R_0_] and the corresponding gauge factor [(R − R_0_)/(R_0_ε)] (Fig. 3i). The gauge factor stabilizes above 2, indicating substantial potential for strain sensing. Moreover, we conducted resistance-strain curve tests on the gel, as depicted in Fig. 3j. The resistance output demonstrates a well-defined step-like response with increasing strain. We performed repeated measurements of the resistance changes at 10%, 30%, and 50% strains, revealing the stability of the resistance changes (Fig. 3k).

**Fig. 4:**
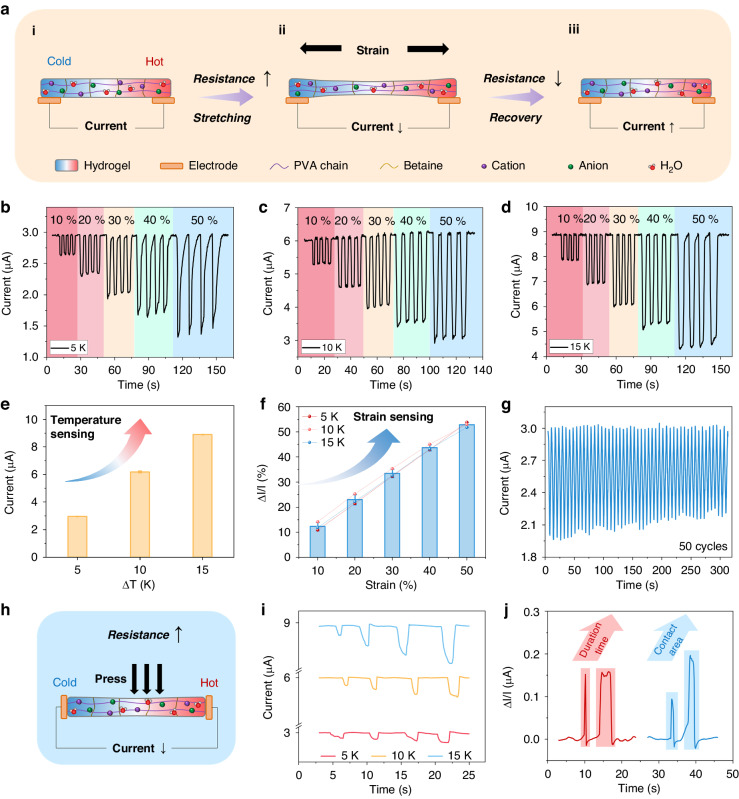
Self-powered temperature/strain sensing of the thermogalvanic hydrogel. **a** Schematic diagrams of the resistance changes before (i), during (ii), and after (iii) stretching. **b-d** Current output after stabilization at 5 K, 10 K, and 15 K temperature differences and current changes at 10%, 20%, 30%, 40%, and 50% tensile strains. **e** Current values at 5 K, 10 K, and 15 K temperature differences. **f** Current variation under 10%, 20%, 30%, 40%, and 50% tensile strains for different temperature differences. **g** Stability testing of the self-powered temperature/strain sensing of hydrogels at fixed temperature difference and strain. **h** Schematic of the resistance change when the hydrogel was pressed. **i** Changes in the current output under a gradually increasing pressure at different temperature differences. **j** Effect of varying the contact time and pressing area on the hydrogel current output

A dual-mode sensing component was crafted by affixing two carbon paper electrodes to the two ends of a slender hydrogel sheet. The temperature- and strain-sensing capabilities of the sensor hinge on the interaction of the redox reactions and a deformable microstructure framework within the hydrogel. When a temperature gradient is established between the two electrodes of the sensor, the reversible redox reactions generate a potential difference in the electrolyte between the electrodes (Fig. 4a(i)). Consequently, if the temperature of one end of the hydrogel sensor is fixed at a known ambient temperature while the other end is in contact with skin, the temperature of the skin surface can be discerned. Moreover, owing to the high environmental stability and good mechanical performance of the composite hydrogel (Fig. 2), when the hydrogel undergoes strain due to an external force, the internal resistance of the hydrogel sensor changes in response to its shape deformation (Fig. 4a(ii, iii)). Considering the correlation between current and resistance, the strain can be gauged by monitoring the change in the current. When temperature and strain are concurrently applied to the hydrogel sensor, the output voltage resulting from the temperature difference not only reflects the temperature but also functions as an internal power source driving strain sensing. By documenting the output current and relative current changes under the influence of a temperature difference, both the temperature of the object in contact with the gel and the strain state of the gel itself can be ascertained. The stretching strain on the skin of the human body resulting from movement is typically below 50%. Therefore, the temperature/strain-sensing performance of the sensor was tested across strain states of 10%, 20%, 30%, 40%, and 50%. During the test, we waited for the current profile varying under the temperature difference to basically stabilize before applying the strain. Figure 4b–d show that under temperature gradients of 5 K, 10 K, and 15 K, the temperature-responsive current of the sensor is well maintained as the thin hydrogel sheet undergoes stretching from 0% to 50% strain. For instance, through the current test curve at a temperature difference of 10 K (Fig. S11), the initial current value in Fig. 4c can be inferred to represent the stabilized value under the temperature difference. The curves in Fig. 4b, d follow the same principle. The temperature of an object can be determined by considering both the room temperature and the temperature difference. In this section, we aim to explore the temperature detection capability by analyzing the output characteristics under various temperature differences, as depicted in Fig. 4b–d. The results show a current of approximately 3 µA at a 5 K temperature difference, approximately 6 µA at a 10 K temperature difference, and approximately 9 µA at a 15 K temperature difference, demonstrating the effective ability of the hydrogel to detect temperature variations. The relative current change induced by strain also exhibits a gradual increase with increasing strain. The current response of the hydrogel to strains of different frequencies is shown in Fig. S12, demonstrating that the main factor affecting the current amplitude is the strain magnitude, whereas the frequency has little effect on it. The response time is shown in Fig. S13, which is also related to the rate of strain generation. Figure 4e shows the output current generated by the gel at various temperature differences when no strain is applied, highlighting the remarkable temperature sensitivity of the gel, with the output current increasing in proportion to the temperature difference. Figure 4f depicts the relationship between the relative current change (ΔI/I, the ratio of the relative current change to the initial current) and strain, indicating a consistent correlation with strain, thus demonstrating the excellent ability of the sensor to perceive strain through its current change response. To assess the stable response characteristics of the current change, tests were conducted under fixed temperature gradients and strain conditions (Fig. 4g). The results revealed a certain attenuation of the current change after 50 cycles, possibly attributable to variations in the gel contact with the electrode during repeated strain and a decrease in the thermoelectric potential over time caused by thermal conduction. While previous tests primarily focused on the transverse strain perception of the hydrogel sheet, the gel also demonstrated the ability to perceive longitudinal strain changes, as exemplified by compression tests (Fig. 4h). To confirm that the output due to pressing is not based on the piezoelectric effect, we pressed each end of the gel and observed the same trends in the voltage changes (Fig. S14), thus verifying that the response is mainly due to the piezoresistive effect. Figure 4i presents the current variation curves with increasing compression strength at different temperature differences. As the temperature difference increases, the steady-state current gradually increases, mirroring the trend observed in the stretching strain perception of the current. The current variations under different compression durations and areas were also tested (Fig. 4j). The current curve exhibits variations and recovery with the application and removal of compression, and a larger compression area results in an increased resistance change, leading to a greater change in the current.

**Fig. 5:**
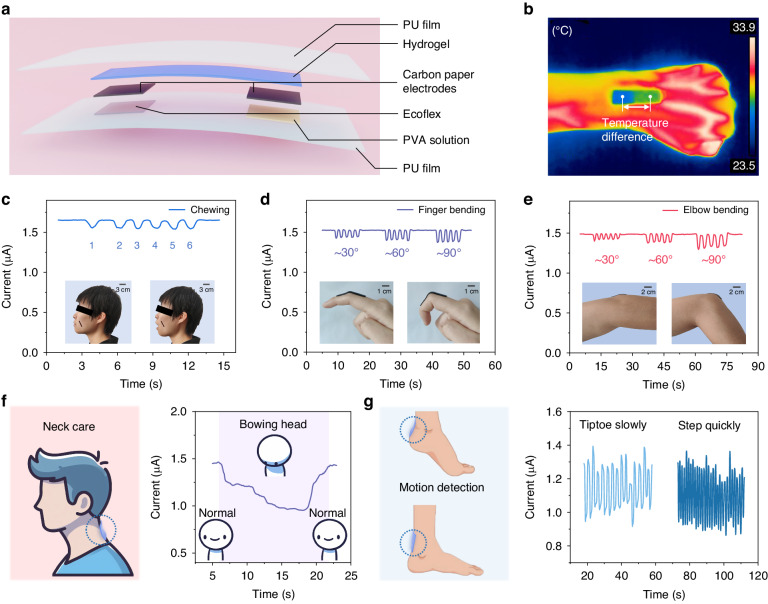
Application demonstration of autonomous temperature and strain sensing by the thermogalvanic hydrogels. **a** Schematic of encapsulation of the hydrogel. **b** Infrared image of a hand showing the visible temperature difference between the ends of the gel sheet. **c**–**e** Current variation curves during chewing, finger bending, and elbow bending. **f** Detection of the head-down degree for neck healthcare. **g** Application of the hydrogel-based e-skin to the foot for monitoring the exercise intensity

We conducted a self-powered assessment of the temperature/strain-sensitive hydrogel for the detection of on-body strain. The gel materials, characterized by nontoxicity, a nonhazardous nature, and a degree of water retention, were encapsulated to enhance the overall performance. The encapsulation structure is delineated in Fig. 5a. Polyurethane membrane (PU)-based dressings, which are recognized for their common use as waterproof dressings that offer transparency, waterproofing, breathability, and bacterial barrier advantages, were employed as the primary material for encapsulation. To optimize the synergy among the gel, carbon paper electrode, and PU substrate and establish a stable temperature gradient across the gel, a PVA solution and Ecoflex served as binders. Ecoflex possesses good thermal insulation properties, which can effectively prevent conduction of human body heat. The effectiveness of the encapsulation in forming a temperature difference was confirmed by capturing images of the device adhered to a hand using an infrared imager (Fig. 5b), revealing a distinct temperature contrast between the two ends of the device. The subsequent text details the performance of the thermoelectric gel in actively sensing temperature and strain on the body skin surface (Fig. 5c–e). The initial current reflects the thermal current output generated by the gel in the presence of a temperature difference between its two ends, with the magnitude indicating the body temperature. In terms of temperature detection, the prepared hydrogel could detect a temperature difference of 0.1 K with a response time of approximately 30 s (Fig. S15a). At a room temperature of approximately 20 °C, it could perceive a temperature of ~37 °C (close to the human body temperature). The hydrogel also exhibited an output response to ~70 °C, but this response was attenuated over time probably due to the higher temperature at one end leading to an unstable temperature difference (Fig. S15b). The lack of response of the thermal voltages to strain suggests minimal interference of strain with the temperature at the ends of the gel (Fig. S16). The application of the gel device to the cheeks facilitated detection of facial muscle movements during chewing (Fig. 5c), with the output curve correlating with each chewing motion. Similarly, gel devices were applied to the finger joint (Fig. 5d) and elbow joint (Fig. 5e). The greater starting current observed for the hydrogel device after thermal equilibration during the mastication test may be attributed to the experimenter’s facial temperature, which could have been higher than that of the hands and arms. As the degree of joint flexion increased, the observed current change exhibited a proportional increase.

We further explored the application of the hydrogel-based e-skin in health and motion monitoring. As shown in Fig. 5f, the gel patch was attached to the neck skin. As the degree of head bowing increased, the hydrogel was gradually stretched, and the current change amplitude increased, whereas the current returned to the initial state after the head was lifted. This application demonstrates the potential to prevent people from bowing their heads for a long time at work. Monitoring of the motion state is shown in Fig. 5g, and from the frequency of the current response, it can be seen that the intensity of the motion increased from tiptoeing to fast stepping. In addition, the thermoelectric gel sheets not only utilize human skin as a heat source for self-powering but also independently sense external stimuli. For instance, as depicted in Fig. S17a, when integrated into a robotic hand, the gel patch enables a cold mechanical carrier to detect an external heat source by utilizing the thermogalvanic effect. The ability of the monolithic gel to pinpoint the location of the heat source and respond to its contact duration were then separately confirmed. The upper panel in Fig. S17b displays the output signals generated when the same heat source approaches one end, the middle, or the opposite end of the gel sheet. The directional outputs indicate that the thermoelectric gel sheet can effectively identify the contact position of the heat source in one dimension. The lower panel of Fig. S17b presents the output signals as the contact time of the heat source with the gel is progressively extended. The output gradually increases, reflecting that the longer the gel stays in local contact with the heat source, the more heat is transferred, leading to a larger temperature difference. In summary, on-body dual-modal temperature/strain sensing provides effective guidance for developing good lifestyle habits and healthcare. Table S1 summarizes a comparison of the properties of the hydrogel-based e-skin in this study with those of other representative hydrogel sensors previously reported, including the stretchability, resistance to desiccation, self-powered features, and input parameters, which illustrates that the prepared hydrogels possess a variety of promising attributes.

## Conclusion

We present a flexible and stretchable hydrogel-based electronic skin for self-powered dual temperature/strain sensing due to its thermogalvanic and piezoresistive effects. The introduction of zwitterions enhances the anti-drying properties of the hydrogel, and the favorable conductivity endows it with a good thermal current output. The responsiveness of the hydrogels to varying temperatures and strains was systematically evaluated, and consistent and predictable trends were observed. We demonstrated the self-powered perception of human body temperature and strain of the encapsulated hydrogel e-skin by applying it to the cheeks, fingers, and elbows, utilizing the difference between the human skin surface and ambient temperatures and the current response caused by strain. Furthermore, the ability to determine the head-down state and activity status is highly meaningful for current lifestyle habits. Through this exploration of the dual-mode self-powered e-skin, we aim to contribute to the advancement of wearable technologies, opening new avenues for real-time health monitoring and human-machine interactions.

### Supplementary information

Supplementary Materials
