# Supplementary material for: Thermogalvanic hydrogel-based e-skin for self-powered on-body dual-modal temperature and strain sensing
Source: Microsyst Nanoeng. 2024 Apr 28;10:55. doi: 10.1038/s41378-024-00693-6 (PMC11055913; doi:10.1038/s41378-024-00693-6)
Supplement: Supplementary file 1 — Supplementary Materials [file 41378_2024_693_MOESM1_ESM.docx]

Supplementary Materials for

**Thermogalvanic hydrogel-based e-skin for self-powered on-body dual-modal temperature and strain sensing**

Zhaosu Wang^1^, Ning Li^1^, Xinru Yang^1^, Zhiyi Zhang^2^, Hulin Zhang^1,^*, Xiaojing Cui^3,^*

^1^College of Electronic Information and Optical Engineering, Taiyuan University of Technology, Taiyuan, 030024, China

^2^College of Materials Science and Engineering, Taiyuan University of Technology, Taiyuan, 030024, China

^3^School of Physics and Information Engineering, Shanxi Normal University, Taiyuan 030031, China

*Corresponding author: Hulin Zhang; Xiaojing Cui

Email: zhanghulin@tyut.edu.cn (H.Z.); 20210084@sxnu.edu.cn (X.C.)


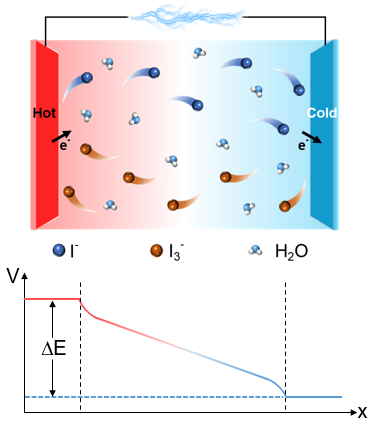


Fig. S1 Voltage distribution of the reversible redox reaction 3I^−^ ⇌ I_3_^−^ + 2e^−^, where ΔE represents the electric potential difference.


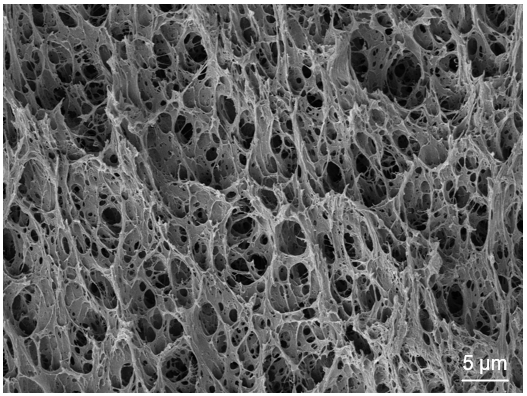


Fig. S2 SEM images of a PVA/Betaine-I^−^/I_3_^−^ hydrogel.


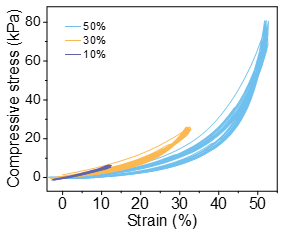


Fig.S3 Tensile cycling of the hydrogels at strains of 10%, 30%, and 50% (30 cycles).


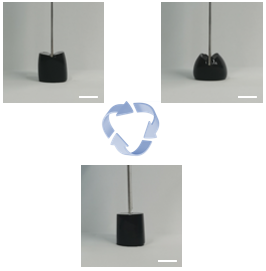


Fig. S4 Pressing the hydrogel with a steel ruler. Scale bar: 1cm.


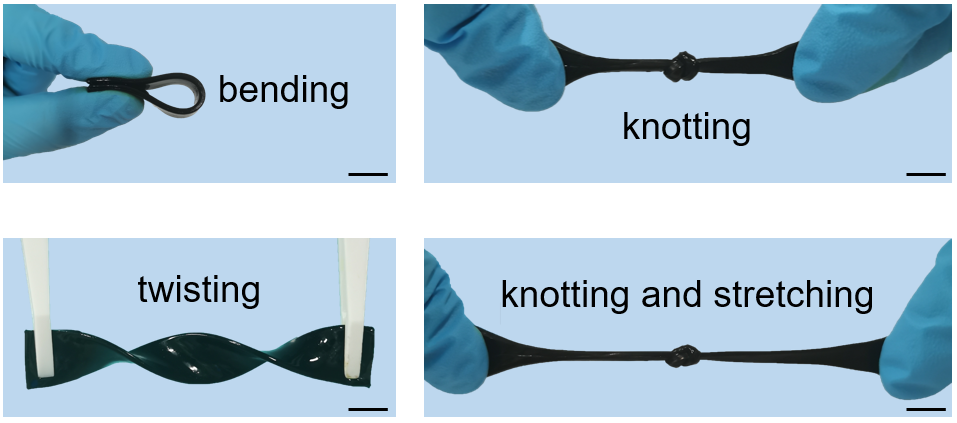


Fig. S5. Physical photos of the hydrogel sheet as it is bent, twisted, knotted and stretched. Scale bar: 1 cm.


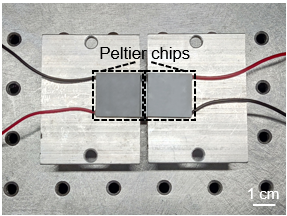


Fig. S6 The constructed temperature gradient platform consists of two Peltier chips and heat sink blocks.


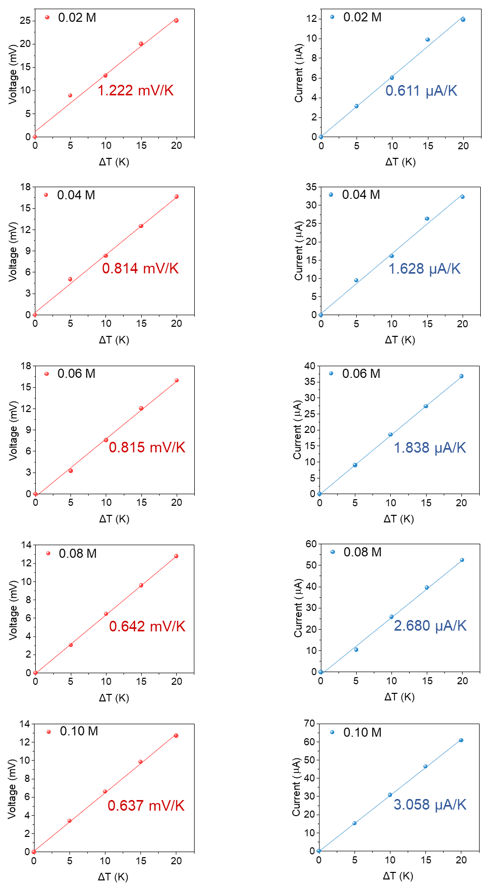


Fig. S7 Output voltage and current of hydrogels with different I^−^/I_3_^−^ concentrations at different temperature differences.


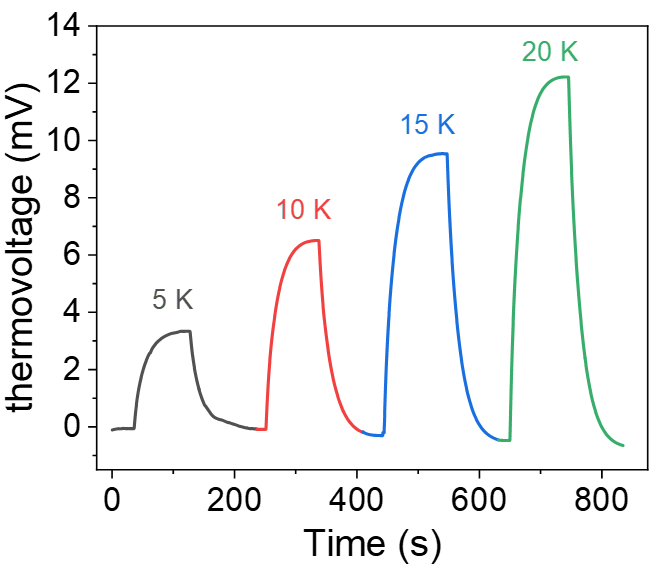


Fig. S8. The thermovoltage-time curve for samples with redox pair concentration of 0.10 M.


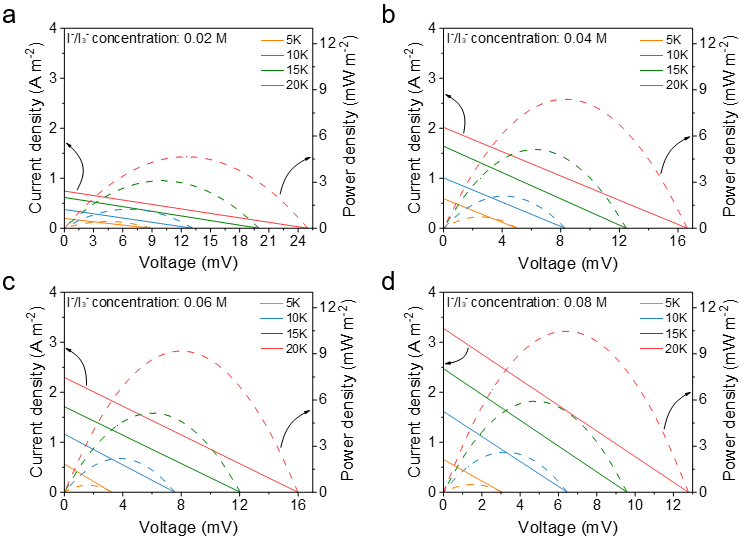


Fig. S9 Analogous voltage-current-power density curves for I^−^/I_3_^−^ concentrations of **a** 0.02 M, **b** 0.04 M, **c** 0.06 M, and **d** 0.08 M.


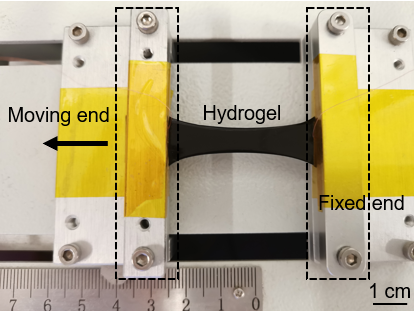


Fig. S10 The controlled strain platform.


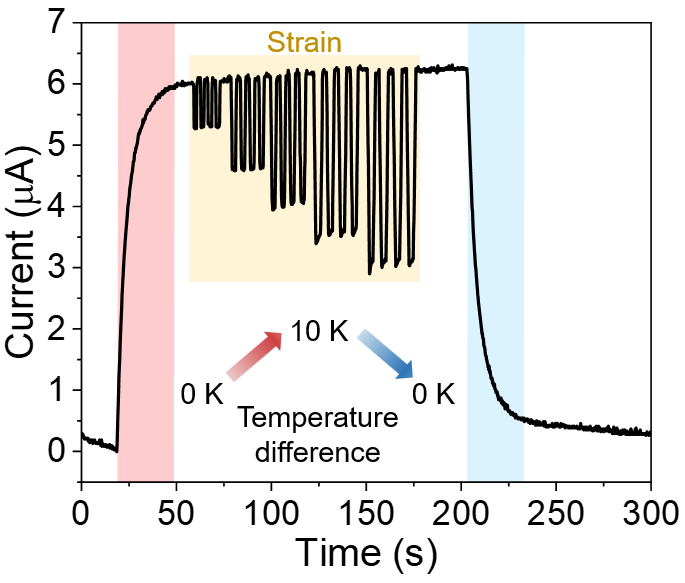


Fig. S11 Current output when a 10 K temperature difference is applied at both ends of the hydrogel then strains are applied and finally, the temperature difference is withdrawn.


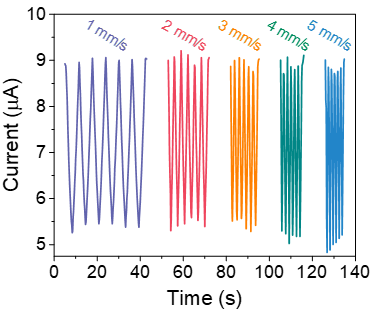


Fig. S12 Current response of the hydrogel to strains of different frequencies.


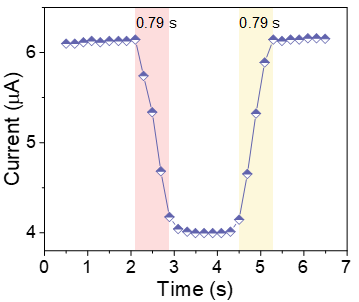


Fig. S13 Current response time of the thermogalvanic hydrogel at 10 K temperature difference and 30% strain.


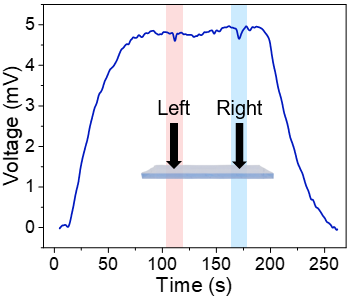


Fig. S14 Voltage response when pressing the left and right end of the hydrogel, respectively.


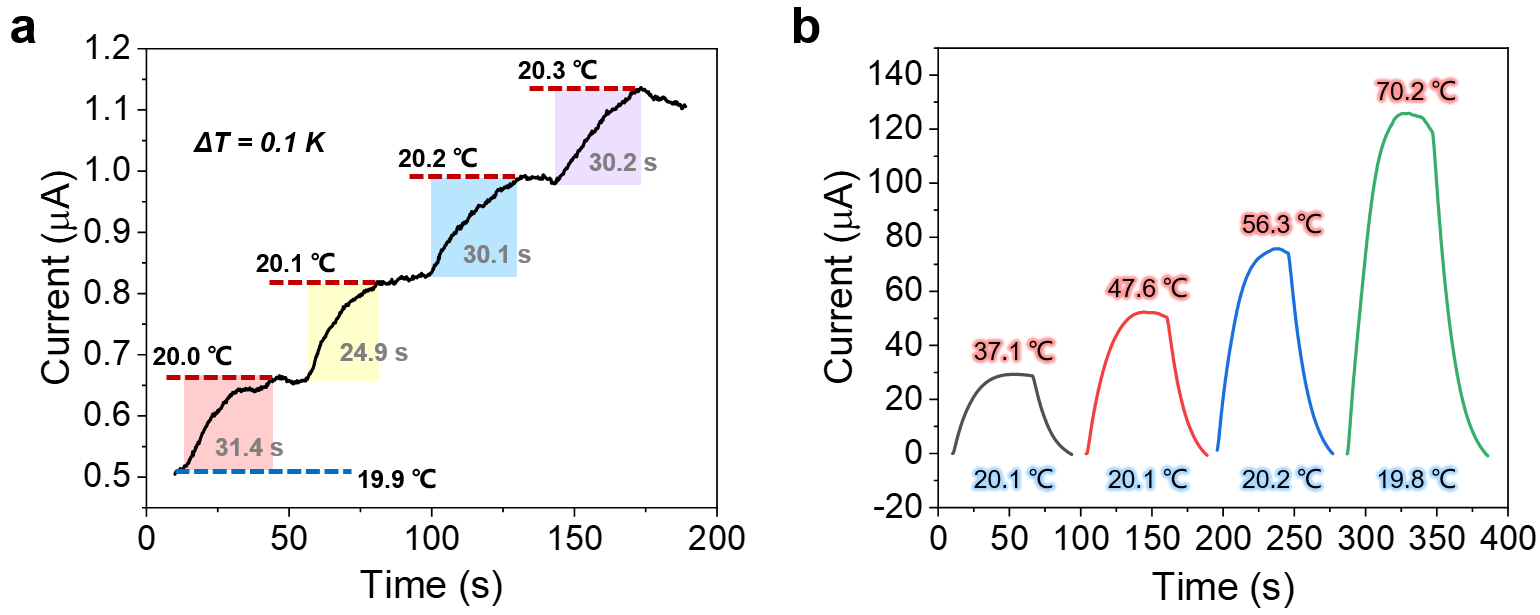


Fig. S15 **a** Response time and detection limit of the thermogalvanic hydrogel. **b** Current output at various temperatures.


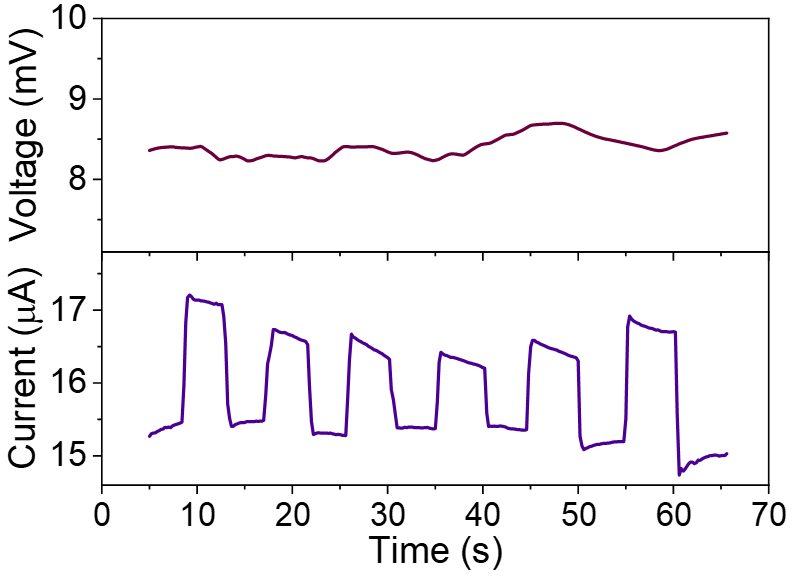


Fig. S16. Voltage and current output during strain action at a fixed temperature difference.


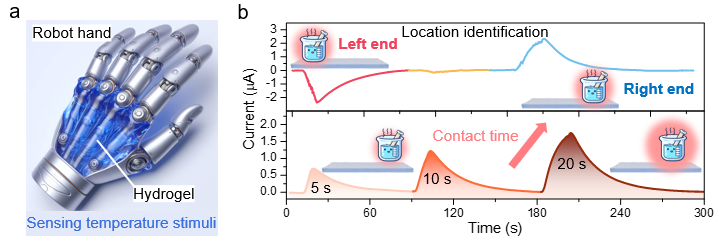


Fig. S17 **a** Schematic diagram of the integration of the hydrogel with a robotic hand for self-powered sensing of external heat sources. **b** Thermoelectric current output when the thermal source contacts the left and right ends of the hydrogel film, and under different durations of thermal contact (5 s, 10 s, 20 s).

Table. S1 A comparison of the hydrogel in this work with other representative reported hydrogel-based sensors in terms of stretchability, anti-drying, self-powered feature and input parameters.

| Stretchability  (%) | Anti-drying | Self-powered | Input Parameters | Ref. |
| --- | --- | --- | --- | --- |
| >600 | Yes | Yes | Temperature, strain | This work |
| 720 | − | No | Temperature, strain | [1] |
| 1044 | − | No | Temperature, strain | [2] |
| >400 | − | No | Temperature, strain | [3] |
| 991% | Yes | No | Temperature, strain | [4] |
| ~600 | Yes | No | Temperature, strain | [5] |
| 760% | − | No | Temperature, strain | [6] |
| ~430 | Yes | Yes | Temperature | [7] |

References:

1. Pang, Q., Hu, H., Zhang, H., Qiao, B. & Ma, L. Temperature-Responsive Ionic Conductive Hydrogel for Strain and Temperature Sensors. *ACS Appl. Mater. Interfaces* **14**, 26536-26547, (2022).
2. Zhang, R. *et al.* Thermoplastic charge-transfer hydrogels for highly sensitive strain and temperature sensors. *J. Mater. Chem. A* **11**, 8320-8329, (2023).
3. Liu, H. *et al.* Fast Self-Assembly of Photonic Crystal Hydrogel for Wearable Strain and Temperature Sensor. *Small Methods* **6**, 2200461, (2022).
4. Ge, G. *et al.* Muscle-Inspired Self-Healing Hydrogels for Strain and Temperature Sensor. *ACS Nano* **14**, 218-228, (2020).
5. Chen, H. *et al.* High toughness multifunctional organic hydrogels for flexible strain and temperature sensor. *J. Mater. Chem. A* **9**, 23243-23255, (2021).
6. Zhao, R., Zhao, Z., Song, S. & Wang, Y. Multifunctional Conductive Double-Network Hydrogel Sensors for Multiscale Motion Detection and Temperature Monitoring. *ACS Appl. Mater. Interfaces* **15**, 59854-59865, (2023).
7. Li, X. *et al.* Thermogalvanic hydrogels for self-powered temperature monitoring in extreme environments. *J. Mater. Chem. C* **10**, 13789-13796, (2022).
